# No Two Workforces Are the Same: A Systematic Review of Enumerations and Definitions of Public Health Workforces

**DOI:** 10.3389/fpubh.2020.588092

**Published:** 2020-11-19

**Authors:** Rory D. Watts, Devin C. Bowles, Eli Ryan, Colleen Fisher, Ian W. Li

**Affiliations:** ^1^School of Population and Global Health, The University of Western Australia, Perth, WA, Australia; ^2^Australian National University, Canberra, ACT, Australia; ^3^Council of Academic Public Health Institutions Australasia, Canberra, ACT, Australia

**Keywords:** public health workforce, systematic review, public health workforce development, global health workforce, benchmarking, public health workforce definition

## Abstract

The delivery and coordination of public health functions is essential to national and global health, however, there are considerable problems in defining the people who work in public health, as well as estimating their number. Therefore, the aim of this systematic review was to identify and explore research which has defined and enumerated public health workforces. In particular, how were such workforces defined? Who was included in these workforces? And how did researchers make judgments about the size of a workforce? In this systematic review, we identified 82 publications which enumerated a public health workforce between 2000 and November 2018. Most workforce definitions were unique and study-specific and included workers based on their occupation or their place of work. Common occupations included public health nurses and physicians, epidemiologists, and community health workers. National workforces varied by size, with the United States and Switzerland having the largest public health workforces per-capita, although definitions used varied substantially. Normative assessments (e.g., assessments of ideal workforce size) were informed through opinion, benchmarks or “service-target” models. There are very few regular, consistent enumerations within countries, and fewer still which capture a substantial proportion of the public heath workforce. Assessing the size of the public health workforce is often overlooked and would be aided by fit-for-purpose data, alignment of occupations and functions to international standards, and transparency in normative methods.

## Introduction

The public health workforce can be regarded as “the stock of all individuals engaged primarily in the improvement of the health of populations” (1). This stock is heterogeneous, and the size and composition of the workforce are difficult to capture. Regardless, it is important to regularly assess size, and to make an assessment about whether that size is appropriate, so that public health responsibilities are optimally met. The importance of the public health workforce has been underscored this year due to the COVID-19 pandemic, along with questions around whether the size and composition of this workforce is appropriate to meet the demands placed on it. Furthermore, an increasingly connected world requires international coordination, and such coordination in public health requires transparency about who is part of this workforce, and what they do.

While there is considerable review literature on the public health workforce, particularly surrounding capacity building for (2–6) and education of the workforce (4, 7–10), there is relatively little which reviews workforce enumeration. To our knowledge, there have been two previous reviews (11, 12), both of which at least in part, reviewed studies which assessed the size of the public health workforce in the United States. Beck and Boulton found relatively few papers which considered the size and composition of the workforce, with one estimate of the US workforce at 450,000 and a few publications looking at epidemiology and public health nutrition workforces. Merrill et al. focused on national enumeration efforts, noting a 10% decline of the public health workforce after 1980 (using the same paper noted in Beck and Boulton to support the finding). Beck and Boulton also looked at papers which forecasted workforce “demand” (12), finding few papers, but papers with a consistent message: a shortage was predicted.

Both reviews are laudable, and this article adds to the breadth of their work. Firstly, by including research efforts outside the United States where public health workforces have been enumerated, so that preliminary comparisons can be made internationally. Secondly, by considering the definitions and occupations used by researchers. Thirdly, by distinguishing between normative and positive workforce enumerations, where a positive enumeration presents an objective statement about the workforce (e.g., the public health workforce has 100,000 workers), whereas a normative enumeration expresses value judgments (e.g., the public health workforce ought to have 120,000 workers). Normative work is often neglected, or conflated with demand, and it is important to distinguish it, and explore the methods which produce it.

Therefore, we formulated the following research questions for this literature review:

1. When researchers enumerate people who work in public health, how do they define the workforce?
2. When researchers enumerate people who work in public health, which occupations do they include for the purposes of enumeration?
3. When researchers enumerate a country's national public health workforce, what is the count of the workforce? How large is this relative to their population?
4. When researchers enumerate a country's national public health workforce, what is the composition of that workforce with respect to occupations?
5. When researchers make a normative statement about the size of a public health workforce, what are the methods they use? What conclusions do they find?

## Materials and Methods

### Eligibility Criteria

#### Inclusion Criteria

The following aspects of the systematic review question formed the inclusion criteria when assessing potentially relevant literature:

##### Population

Must consider all or part of the public health workforce, using the following definition by Rotem et al. “(those who are) engaged in activities related to the protection (promotion and/or restoring) of the collective health of whole or specific populations [as distinct from activities directed to the care of individuals (13)].”

##### Enumeration

Must include a positive or normative assessment of workforce size e.g., a count of workers, count of full-time equivalents (FTE).

##### Publication Year

We considered all publications published from 1 January 2000 until 1 November 2018.

##### Language

We considered English language publications. If an English abstract was available, but the full-text version was not entirely in English, we determined on an individual basis whether the available text had enough information for our data collection purposes.

##### Publication Type

We considered peer-reviewed articles, theses, books, conference abstracts, and reports. We also included secondary sources (a publication which reported the results of an enumeration) if the primary source was not available.

#### Exclusion Criteria

We excluded certain media, such as newspaper and magazine articles. We also excluded publications where enumeration was not a research objective, but partially observed (e.g., sample size in a survey).

### Information Sources

#### Search Terms

Scoping was undertaken to identify suitable search terms and search strategies to be included. We adopted four search strategies to minimize missing publications, which were a combination of keywords and MeSH terms relating to workforces (e.g., “epidemiologists”), size features and methods (e.g., “enumeration”) and workforce qualifiers [e.g., “health workforce”(MeSH)]. Full search strategies are featured in the Supplementary Material.

#### Databases and Gray Literature

Three databases were used in the review: PubMed, ProQuest, and Web of Science *via* ISI. All results were screened. Two search engines were used to identify relevant gray literature, Google Scholar (www.scholar.google.com), and ScienceDirect (www.sciencedirect.com). Both search engines were searched using the terms “Public Health Workforce” and “‘Public Health Workforce’ AND ‘enumer^*^.”’ Sorted by relevance, the first 200 hits were screened for inclusion. We also conducted a second gray literature search to find national estimates of total public health size. Google was searched for each country which appears in the United Nations World Population Prospects (14) and appended to the country was the term “Public Health Workforce” (e.g., Australia Public Health Workforce). The first 50 results were screened for relevance and any potentially relevant publications or websites were reviewed in full.

#### Study Selection

Study selection according to the pre-defined inclusion criteria proceeded according to a two stage, hierarchical process: titles and abstracts firstly, and full texts secondly. Eligibility assessment was performed independently in an unblended standardized manner by one reviewer (RW) and doubts over the relevance of publications were assessed by a second reviewer (DB). Uncertainty over the presence of a relevant inclusion criterion led to articles being retained for assessment of the full text. As titles and abstracts in gray literature may not conform to peer-reviewed publication standards, their full-text was assessed if deemed relevant. Following screening, full-text review and data collection took place using Zotero and Microsoft® Excel. Full articles were obtained in PDF or full-text HTML format.

#### Snowball Sampling

We conducted both forward (looking at papers which had cited the article) and backward (looking at papers which the article had cited) snowball sampling on papers for full text screening using Web of Science and PubMed. We did not sample further than one level of citation (i.e., we did not look at citations of citations).

### Data Collection Process

#### Data Items

Data extraction was undertaken in Microsoft® Excel and were decided upon through initial scoping of the literature and discussion within the review team. Data extraction was pilot tested on 10 full text articles and refined according to assessment of the relevancy of variables extracted. We collected the data under the following headings: country/location, workforce studied, workforce definition(s), positive/normative assessment used, assessment methods, and assessment findings.

#### Alignment of Occupational Categories

For research questions which considered the occupations of workers (questions 2 and 4), we mapped the stated occupations to the most relevant occupational category in the International Standard Classification of Occupations (ISCO-08) (15). Most classifications were straightforward, but a small number of occupations could have been classified as more than one category. These classifications were discussed amongst the research team and our reasoning, as well as the mapping rules for each classification are included in the Supplementary Material.

#### National Workforce Estimates

Total national workforces were converted to “workers per 100,000” using the United Nations population estimates for the year in which the estimate was taken [data for England was obtained using national sources (16)]. For countries with more than one estimate, only the most recent was included.

## Results

### Study Selection and Characteristics

**Figure 1:**
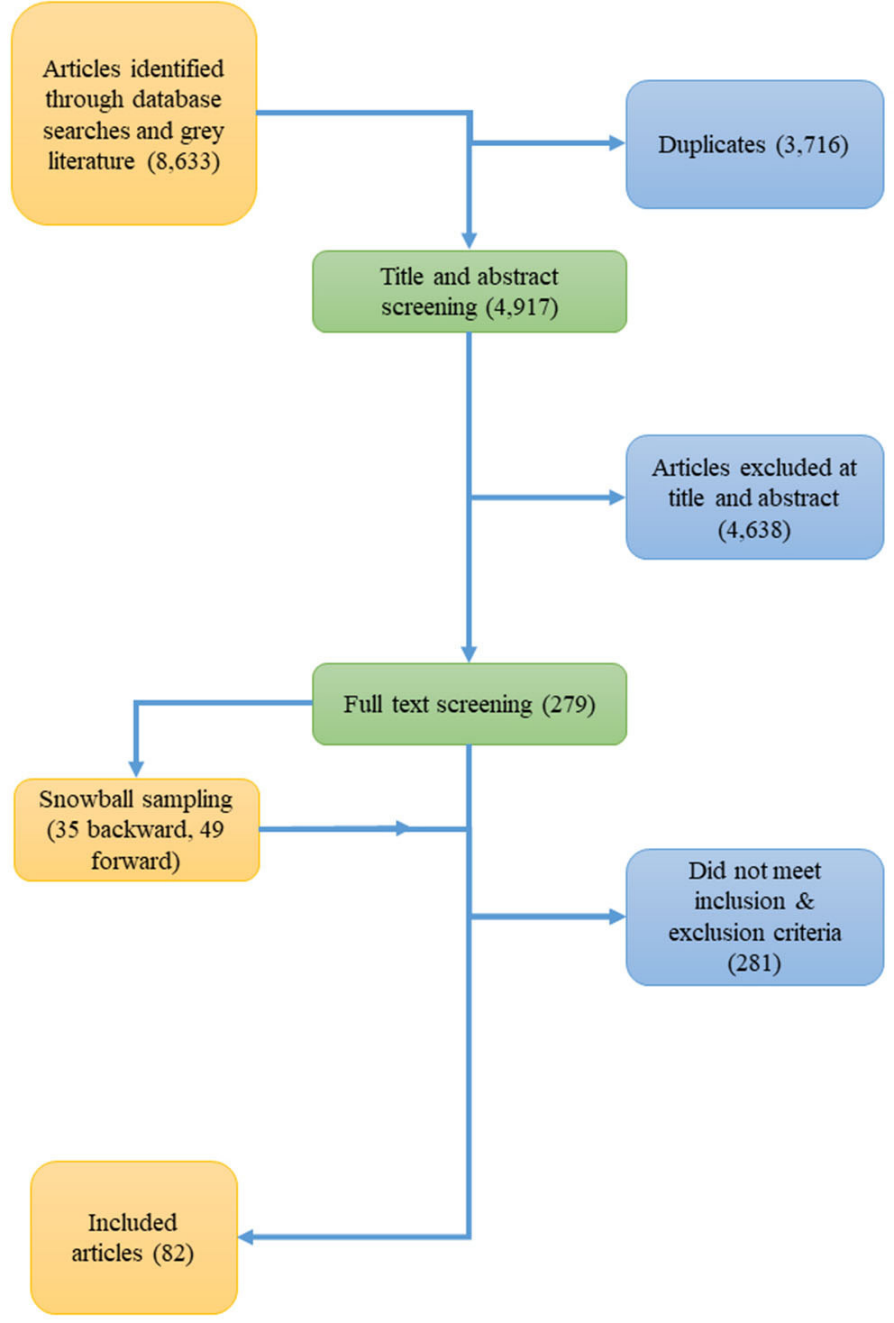
PRISMA flow chart showing flow of information from article identification to inclusion.

Figure 1 shows a PRISMA flow chart from initial yield to our final search results. Using our search strategies, we initially yielded 8,633 entries. There was substantial overlap between strategies, and after filtering for duplicates, we identified 4,917 unique articles for title and abstract screening. Two hundred and seventy nine obtainable articles were reviewed, and snowball sampled for other relevant articles yielding an additional 35 items for review with backwards searching, and 49 items with forward searching. Two hundred and eighty one items were excluded because they did not satisfy the inclusion criteria or met the exclusion criteria, therefore a total of 82 publications were included in the review. Overall, publications appear to be increasing over time. Publications considering workforces in the United States accounted for over a third of publications (37.6%), followed by the United Kingdom, Australia, and New Zealand (4.95% each). The reference list of included publications appears in the Supplementary Material.

### When Researchers Enumerate People Who Work in Public Health, How Do They Define the Workforce?

Workforces varied in scope, from highly specific occupations (e.g., food safety epidemiologists) to disparate groups of people who provided a key function of public health [e.g., anyone with the opportunity or ability to positively impact health through their work (17)]. In terms of defining a workforce, publications provided a pragmatic definition (i.e., they articulated exactly who would or would not be included in the enumeration), a conceptual definition (i.e., they articulated an abstract idea about who the workforce consisted of), or both. Almost all definitions were pragmatic in nature, with conceptual definitions commonly included as a survey item to help workers self-identify as part of the public health workforce (18). Pragmatic definitions could generally be considered as defining a workforce on the basis of: employer type (e.g., employees of a local health center and members of a college of physicians); what functions the workers performed (e.g., all workers who perform environmental health functions); the workers' occupations (e.g., all epidemiologists and all persons who identified as part of the public health nutrition workforce); or the workers' prior training (e.g., all persons with a Public Health degree). When conceptual definitions were used, no two were identical but commonly focused mainly on the core ideas “population” and “health” [e.g., “anyone who works with groups and/or communities to protect, promote, or advance health/wellness” (19)]. Some researchers also delineated the workforce on the basis of whether the work was a primary duty, and one study included only workers who performed functions for ≥0.5 h per week (20).

### When Researchers Enumerate People Who Work in Public Health, Which Occupations Do They Include for the Purposes of Enumeration?

Table 1 presents the frequency with which occupational categories were considered in the publications we found. Nurses were the most commonly considered category in the public health workforce, followed by epidemiologists (as part of ISCO-08 category 2131), community health workers and specialist medical practitioners (most commonly the “public health physician”). A total of 33 four-digit ISCO-08 categories were mentioned in publications, with the majority belonging to the “health professionals” category but with considerable mention of managerial, administrative, and research categories as well.

**Table 1:** Frequency of occupations mentioned by researchers.

**ISCO-08 Category**	**Frequency of mentions** **(% of all mentions)**
2221—Nursing Professionals	45 (12%)
2131—Biologists, Botanists, Zoologists and Related Professionals (incl. Epidemiologist)	35 (9%)
3253—Community Health Workers	34 (9%)
2212—Specialist Medical Practitioners (incl. Public Health Physician)	27 (7%)
2265—Dieticians and Nutritionists	21 (6%)
2263—Environmental and Occupational Health and Hygiene Professionals	21 (6%)
1342—Health Services Managers	21 (6%)
2261—Dentists	19 (5%)
2635—Social Work and Counseling Professionals	18 (5%)
3212—Medical and Pathology Laboratory Technicians	16 (4%)
3343—Administrative and Executive Secretaries	15 (4%)
3252—Medical Records and Health Information Technicians	15 (4%)
2211—Generalist Medical Practitioners	14 (4%)
2120—Mathematicians, Actuaries and Statisticians	11 (3%)
1219—Business Services and Administration Managers Not Elsewhere Classified	5 (1%)
2250—Veterinarians	5 (1%)
3257—Environmental and Occupational Health Inspectors and Associates	5 (1%)
1211—Finance Managers	4 (1%)
2143—Environmental Engineers (incl. Sanitary Engineers)	4 (1%)
2631—Economists	3 (<1%)
2422—Policy Administration Professionals	3 (<1%)
2634—Psychologists	2 (<1%)
2632—Sociologists, Anthropologists, and Related Professionals	2 (<1%)
2262—Pharmacists	1 (<1%)
4131—Typists and Word Processing Operators	1 (<1%)
2222—Midwifery Professionals	1 (<1%)
3423—Fitness and Recreation Instructors and Programme Leaders	1 (<1%)
2310—University and Higher Education Teachers	1 (<1%)
9112—Cleaners and Helpers in Offices, Hotels and Other Establishments	1 (<1%)
2113—Chemists	1 (<1%)
2269—Health Professionals Not Elsewhere Classified (incl. health experts)	1 (<1%)
NEC—Not elsewhere classifiable	21 (6%)

### When Researchers Enumerate a Country's National Public Health Workforce, What Is the Count of the Workforce? How Large Is This Relative to Their Population?

We found seven publications reporting estimates for 11 national public health workforces. Both the highest absolute estimate and estimate per 100,000 persons was for the United States (21), with a total workforce of 326,602 (104.2 per 100,000) in 2012. Switzerland (22) had the second highest workforce per 100,000 workers (102.6 per 100,000) in 2013, and the lowest amount of workers per 100,000 population was Eritrea [7.3 per 100,000 (23)] in 2015. The country average (mean workers per 100,000) and the population average (total workers/total population) were 62.7 and 81.5 workers per 100,000 respectively. These estimates are available in Table 2.

**Table 2:** National public health workforce estimates of 10 countries, ordered from smallest to largest ratio of workers per 100,000 population.

**Country**	**Reference year**	**Workforce size**	**Population in reference year**	**Workers per 100,000 population**	**References**
Eritrea	2015	352.8	4,846,976^a^	7.3	(23)
Germany	2000	20,810.00	81,487,757^a^	25.5	(24)
Italy	2015	26,435	60,800,000^a^	43.5	(24)
Poland	2017	17,080.00	38,170,712^a^	44.7	(24)
Moldova	2015	2,323.00	4,065,980^a^	57.1	(24)
Slovenia	2015, 2016	1,203.00	2,074,788^a^	58	(24)
England	2014	38,500.00	54,300,000^b^	70.9	(25)
Netherlands	2012	12,000.00	16,789,095^a^	71.5	(26)
New Zealand	2004	3,600.00	4,088,000^a^	88.1	(27)
Switzerland	2013	8,342.00	8,132,674^a^	102.6	(22)
United States	2012	326,602.00	313,335,423^a^	104.2	(21)

Total workforce represents the “best estimate” from the reference e.g., the median estimate of the “best guess” scenario. Population in reference year taken from

a *United Nations et al. (14)*.

b *Office for National Statistics (16)*.

### When Researchers Enumerate a Country's National Public Health Workforce, What Is the Composition of That Workforce With Respect to Occupations?

**Figure 2:**
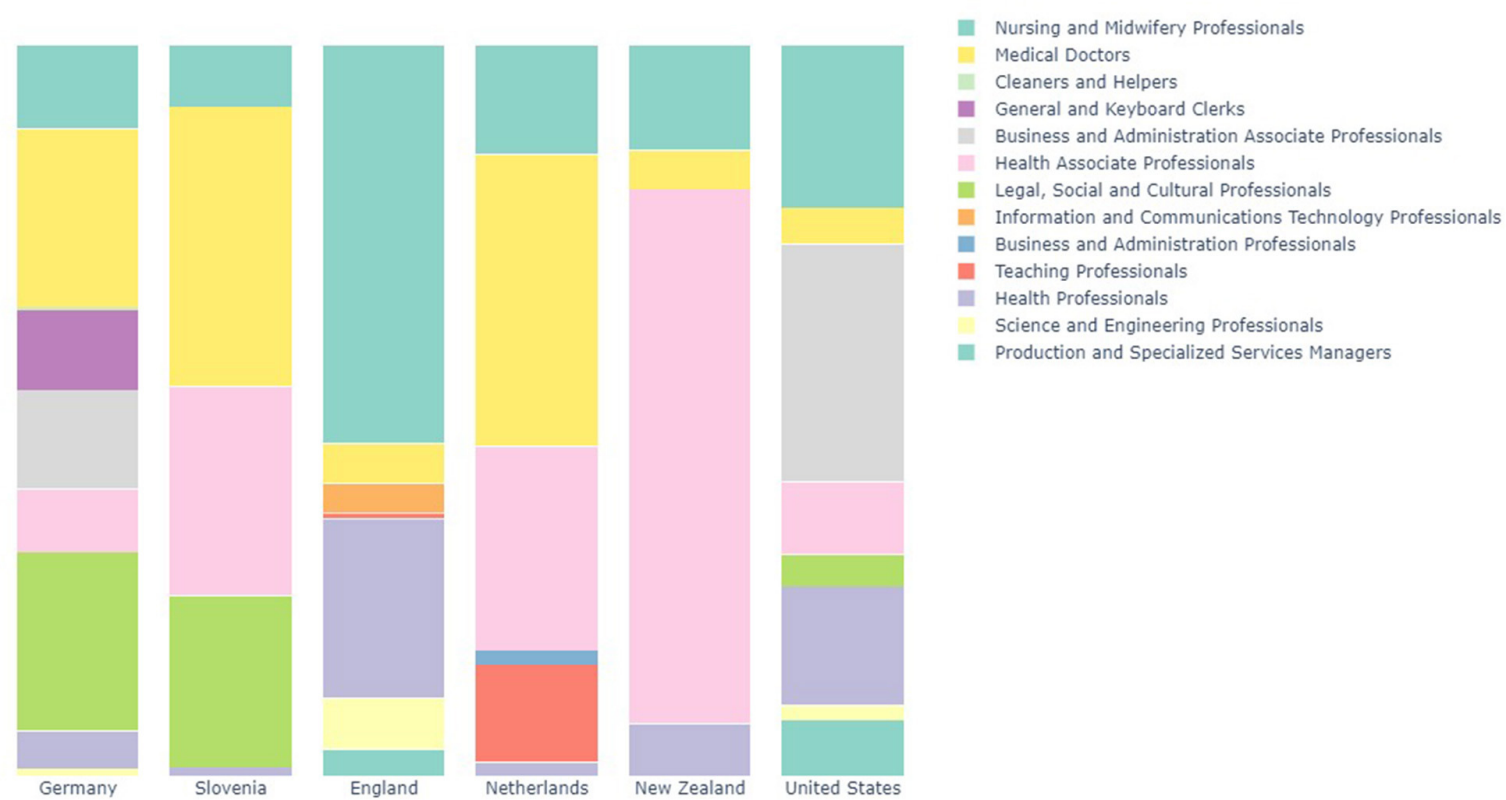
Bar chart showing relative proportions of occupations in National Public Health Workforces.

Figure 2 shows the composition of ISCO-08 two-digit occupations which are present at national level enumerations of public health workforces. All countries identified showed a substantial proportion of nursing and midwifery personnel in the public health workforce, ranging from 8 to over 50% of these workforces. Medical doctors also contributed a large proportion, ranging from 5 to 40% of the total workforce. “Health Associate Professionals,” was the most common aggregated occupation (ISCO-08 code 32) which was primarily composed of health promotion workers (such as in the case of New Zealand) and community health workers. The disaggregated (ISCO-08 four-digit codes) is available in the Supplementary Material.

### When Researchers Make a Normative Statement About the Size of a Public Health Workforce, What Are the Methods They Use? What Conclusions Do They Find?

We found 25 studies which made explicit normative assessments of workforce size, sometimes referred to as workforce “demand” or “need.” A taxonomy of methods used by researchers is available in Table 3. The implication of most normative assessments, whether explicit or not, is to provide an estimate of how many workers there ought to be to provide public health services. However, there were different approaches to providing this estimate including: benchmarking the workforce relative to themselves (e.g., the US workforce relative to their size in 1980); benchmarking the workforce relative to other workforces (e.g., comparing the EU workforce relative to the US workforce in 1980); relying on expert opinion to assess optimal capacity; relying on health system indicators (e.g., % increase in health expenditure) to dictate growth in the workforce; and estimating FTEs required to provide all the services which are said to be provided.

**Table 3:** Approaches to normative assessments of public health workforce size, and an example of the approach from identified publications.

**Normative assessment approach**	**Example of approach**	**References**
Expert opinion	A survey asking state epidemiologists to estimate the ideal number of epidemiologists needed to fully meet epidemiology and surveillance capacity.	(28)
Benchmark using expert opinion	Estimating the number of public health physicians needed in Canada, using a United States estimate based on a committee's expert opinion.	(29)
Benchmark using historical workforce data	Estimating the total number of health service managers in India using historical estimates of health services managers in the United States.	(30)
Benchmark using current workforce data	Using current workforce size per region in the United Kingdom: estimating how many workers would be needed if all regions had the same amount of workers as the region with the most workers.	(18)
Benchmark using other indicator	Using the growth of health expenditure to predict what size of the workforce should be. For example, if health expenditure grows by 4%, so too should the health workforce.	(31)
Service-target based model	Using existing guidelines for public health, nursing and medicine: calculating the total demand that would be generated if all the services were delivered at the level of quality in these guidelines, and the person-time required to provide these.	(32)

When examining the conclusions that researchers came to, the results were nearly unanimous: more workers are needed. Of the 25 studies which made some normative assessment of optimal workforce size, only one study (looking at the public health physician workforce in Australia) projected an excess supply of workers (33).

## Discussion

### Summary of Evidence

We identified 82 publications which assessed the size of public health workforces, with 25 of these making normative assessments about what the present or future workforce size should be. Given the subject matter, it is no surprise that our findings are heterogeneous: there was a range of workforces, settings, and definitions. Despite this, most research which made a normative assessment was quick to identify the gaps between currently supply, and what supply ought to be.

The first notable finding is the sheer volume of studies from the United States, many of which have been identified previously (12). It is evident that there are well-organized, sustainable attempts to profile existing workforces in the United States, and this is encouraging. For example, the Association of State and Territorial Health Officials (ASTHO), and the National Association of County & City Health Officials (NACCHO) both produce regular reports of their workforces (ASTHO has produced four volumes since 2007, NACCHO has produced eight volumes since 1989). There are clear benefits for having such a work stream owned by a large, fixed organization: coverage of the necessary workers, sustainability, and repeatability. While national enumerations are important, we note that we found no studies which looked beyond the national workforce. Although difficult, an international enumeration would allow researchers to investigate the workforce's contribution to global health outcomes and disparities.

We found a variety of workers which constituted the public health workforce, with some workers being unique to the nation where the work was undertaken (e.g., village doctors) (34). Indeed, the organization of a health system plays a particularly important role in which occupations will be considered part of the public health workforce. A particularly explicit example of this is The Republic of Moldova, where legislation has determined the functions and responsibilities of the public health service and therefore the size and composition of the workforce (24). This is very different to other ideas about the public health workforce, such as the very broad definition of the ‘wider public health workforce’ described by the Center for Workforce Intelligence in the UK (17). This poses a problem to providing a pragmatic definition of the public health workforce internationally: there is no definitive set of occupations which can be called the public health workforce with confidence. Our findings suggest some occupations which were common, such as certain nursing specialties (e.g., public health nurses), epidemiologists, community health workers, and public health physicians. Although all these occupations are common, their composition varies drastically between countries, posing questions about the comparability of these workforces, or the transferability of skills in these workforces.

Given the finite set of functions that public health workforces perform (35), there must be substantial overlap between many of these workers (e.g., between various nursing roles such as community health, public health, school nurses, and health visitors), and thus it seems appropriate (although idealistic) for researchers to use conceptual definitions that focus on those who perform public health functions rather than occupations exclusively. However, occupations are important, and we would recommend that when occupations are listed, effort is made to align them with international standards, such as ISCO-08. We would also recommend that future researchers make efforts to describe the functions that are performed by workers. The World Health Organization describe 10 Essential Public Health Operations (EPHOs) (35). Work which aligns the occupations examined to the operations performed would help identify gaps in capacity in a systematic and sustainable way.

We observed that definitions to capture workforces were almost never alike. When definitions were alike, they were being used by the same researchers, for the same group [e.g., the Centers for Disease Control and Prevention's Assessment of Epidemiology Capacity in State Health Departments (36)]. This finding hints at an underlying issue: the relative absence of fit-for-purpose public health workforce data. A substantial number of publications were independent studies conducting cross-sectional surveys to capture a workforce and therefore, they employed an pragmatic definition intended for their purposes, rather than drawing from existing data. However, this issue is not easily fixed for two reasons: many groups of workers perform public health functions, and many of these are not counted regularly.

Which definitions are the most useful? We note that most pragmatic definitions identified workers either by who they were, where they worked, what they did, or what they were taught. As a first step, definitions should be guided by standard research practice i.e., they should provide enough information that another researcher could replicate the work. However, a more critical question is whether these definitions capture all those who should be captured. Of the four aforementioned workforce distinctions (who they are, where they work, what they do, and what they were taught), the most appropriate definition for the public health workforce relates to persons who perform public health functions, i.e., “what they do.” This is due to the nature of public health work being dispersed, in where it is performed and who performs it. However, this is also the most difficult approach. Jambroes et al. (20) provide a useful example for other researchers seeking to characterize a workforce based on World Health Organization EPHOs. We recommend that researchers describe as much about the workforce as possible, including functions performed, occupations, responsibilities and education. A tool which may assist researchers with this is the taxonomy created by Beck et al. (37).

Despite substantial differences in definitions, we have presented a cross-country comparison of estimated national public health workforces, illustrating the composition of ISCO-08 occupations when those roles were possible to infer. We note the difficulties in making any valid comparison, but believe it is an important first-step in counting the total international public health workforce. The United States has the largest public health workforce per-capita, closely followed by Switzerland. Depending on the definition, there are also outliers, such as Eritrea which is an outlier in terms of workforce per capita, or Germany, who have a low workforce per capita relative to other metrics one could consider [e.g., gross domestic product, health spending (38), doctors and nurses per capita, and environmental burden of disease (39)]. Workforce size closely correlates with total population, but should this be the case? Public health workforces provide services which target multiple people at a time, and we might expect to see economies of scale, and therefore a plateauing of workforce size with increasing populations. It may be that most workforces are providing services at the individual/group level (e.g., frontline workers providing vaccines), but more granular enumeration efforts would help us see whether this is the case.

Making normative assessments about workforce size is difficult. Evidence supports the relationship between public health services and the improvement of health outcomes (40–42) but relating these outcomes to an ideal workforce size is a complex task. Having said this, some observed approaches appear more reasonable than others, as they increase transparency and decrease reliance on opinion. The use of benchmarks in particular appears to have the most potential for misuse, as the origins of, and the methods for producing the benchmark (e.g., a benchmark based on opinion, or historical data) are seldom described clearly. Much of the time, use of benchmarks did not have regard for the context in which the benchmark was created. This includes examples where researchers have used benchmarks based on other country's health systems, without due consideration of the country's health context (e.g., stating a shortage in workers in the EU based on a US benchmark). This also includes examples which reference a time period which seems inappropriately distant from today, including the benchmark of 220 public health workers per 100,000 population, which was established by the reporting of workforce numbers in a US congressional report in 1980 (43) and referencing “international benchmarks” for the public health nutrition workforce, citing a book published in 1990 (44). Much has changed about the world and public health in the past several decades, and the use of such benchmarks may not be as appropriate as it once was.

In contrast to the use of benchmarks, the “service-target” model is a more transparent method of assessment, which asks “what do public health workers need to do and how long will it take them?” In two identified studies (32, 45), need was assessed by what a workforce was obliged to do, either through best practice guidelines, or nationally guaranteed packages of care. Estimates of person-time were still reliant on opinions, but these were often made more robust through multiple informants. While this method increases transparency and decreases reliance on opinion, this method falls short when tasks and duties are hard to estimate, and some essential public health functions (e.g., health promotion and advocacy) are hard to estimate. Another important point is that normative estimates may be hard to replicate internationally if they are reliant on national obligations, and therefore it would be difficult to provide a normative estimate of the total international public health workforce using these methods. National obligations can be implicit or explicit, and are likely related to the level of a country's economic development, and their investment in health. There are many other methods which can be used to make normative assessments of workforce size (46) and consideration of these in future research may do well to circumvent some of these issues.

Nearly all researchers who made a normative assessment of workforces came to the same conclusion, regardless of the workforce examined or the country: there is or will be a shortage. The ubiquity of these conclusions is serious, but this seriousness is at times marred by the methods used to arrive at the conclusion. As discussed above, if the benchmark is the highest-ever recorded instance of a workforce, then researchers are more likely than not to find a gap. One particularly obvious example of this involved benchmarking all local health authorities against the local health authority with the highest number of personnel. The conclusion that there was a shortage of personnel given this method is unsurprising. Such conclusions conflate a smaller number in a workforce with a shortage in that workforce. A shortage implies an inability to meet demand, whether that demand is imposed by consumers, or that demand is self-imposed by a set of institutional obligations. Researchers who make such conclusions should explain how the current supply is a shortage, rather than just a decrease in workforce.

### Limitations

The public health workforce literature is dispersed in the literature, gray literature, and websites (some of which have become unavailable since they were originally uploaded). Therefore, we may have missed key sources, although we minimized this by including secondary sources when a primary source could not be found. We may have also missed important keywords, but we minimized this by having all four reviewers contribute their expertise into populating the keyword list, by creating multiple search strategies, and by adopting a snowball search strategy. We note that Beck and Boulton (12) also expressed similar limitations with their keyword search. It is also not clear that our group's intuitions about workforces which fit the provided definition used in this review are appropriate in all national contexts, which is why we included some workforces which were unique and perhaps outliers [e.g., village doctors in China, (34) or the health services research workforce (31, 47)]. Finally, the review included articles written in English, so we have had to rely on the translations of authors working in countries in which English is not spoken. This could feasibly have led to some articles about the public health workforce not using the correct terminology and therefore not being included in the review. It could also result in lack of clarity around the boundaries of the public health workforce. We do not believe that these issues have substantially altered the main conclusions of our work as most of the articles were written by authors in which English is the primary language or is widely understood.

### Conclusion

We have added to the review literature which considers the public health workforce by examining publications which consider the size and definitions of public health workforces. Our work is strengthened through our broad search terms, international scope, and by distinguishing studies which declare what size the workforce is, and what size it should be. To assess whether the size of a public health workforce is appropriate, reliable comparisons must be made, both in terms of comparisons between countries, and comparisons against a normative benchmark. This is difficult to achieve when definitions of workforces disagree, and the methods to benchmark workforces are opaque. By aligning the occupations and functions of workers, and by describing the methods used to make normative assessments, international comparisons of such workforces will be made more easily.

Within the literature, we have identified a lack of clarity when researchers define which workers they are looking at and what functions they perform. As it stands, current national public health workforce estimates are hindered by this lack of clarity. Finally we also found a variety of methods by which researchers make normative assessments about the size of a workforce, many of which lacked transparency and may not be appropriate for the conclusions they produce. Therefore, we make the following recommendations for future researchers:

1. When researchers investigate the public health workforce, effort should be made to describe the occupation, functions performed, responsibilities, and education of this workforce. This will enable more accurate comparisons to be made in the future.
2. Researchers should align occupations to international standards (e.g., ISCO-08) and describe functions performed in terms of Essential Public Health Operations. This will increase transparency and comparability over time.
3. Researchers who make some claim about how large a workforce ought to be must be clear about the methods they use to arrive at such a claim. A shortage is not defined by a reduction in the size of a workforce, but a difference between the supply of workers and the services they are obligated to provide.
4. One transparent and practical normative method is to describe the services which the workforce is obligated to provide, and estimate how many people are required to meet those obligations. We would recommend this rather than using benchmarks. The limitations of such a method may be strengthened by considering this method alongside other methods, such as trend analyses or expert opinion, provided these methods abide by recommendation 3.

## Data Availability Statement

The original contributions presented in the study are included in the article/Supplementary Material, further inquiries can be directed to the corresponding author.

## Author Contributions

ER, DB, and RW: inception. RW, DB, IL, CF, and ER: data collection and design. RW and DB: data analysis. RW: writing. RW, DB, IL, and CF: drafting. All authors contributed to the article and approved the submitted version.

## Conflict of Interest

The authors declare that the research was conducted in the absence of any commercial or financial relationships that could be construed as a potential conflict of interest.
