# Supplementary material for: No Two Workforces Are the Same: A Systematic Review of Enumerations and Definitions of Public Health Workforces
Source: Front Public Health. 2020 Nov 19;8:588092. doi: 10.3389/fpubh.2020.588092 (PMC7711128; doi:10.3389/fpubh.2020.588092)
Supplement: Supplementary file 1 [file Table_1.docx]

1. AlBaker AA, Al-Ruthia YSH, AlShehri M, Alshuwairikh S (2017) The characteristics and distribution of dentist workforce in Saudi Arabia: A descriptive cross-sectional study. Saudi pharmaceutical journal : SPJ : the official publication of the Saudi Pharmaceutical Society 25:1208–1216
2. Alcalde-Rabanal JE, Nigenda G, Bärnighausen T, et al (2017) The gap in human resources to deliver the guaranteed package of prevention and health promotion services at urban and rural primary care facilities in Mexico. Human resources for health 15:49
3. Arrazola J, Binkin N, Israel M, et al (2018) Assessment of Epidemiology Capacity in State Health Departments - United States, 2017. MMWR Morb Mortal Wkly Rep 67:935–939
4. Association of State and Territorial Health Officials (2008) Profile of State and Territorial Public Health Volume One. Arlington, VA
5. Association of State and Territorial Health Officials (2010) Profile of State and Territorial Public Health Volume Two. Arlington, VA
6. Association of State and Territorial Health Officials (2014) Profile of State and Territorial Public Health Volume Three. Arlington, VA
7. Association of State and Territorial Health Officials (2017) Profile of State and Territorial Public Health Volume Four. Arlington, VA
8. Bacon S, Orchard C, Milne R (2007) Specialist capacity in public health: are we hitting the target? Public Health 121:148–153. doi: [10.1016/j.puhe.2006.05.027](https://doi.org/10.1016/j.puhe.2006.05.027)
9. Beck AJ, Boulton ML (2015) Trends and Characteristics of the State and Local Public Health Workforce, 2010–2013. Am J Public Health 105:S303–S310. doi: [10.2105/AJPH.2014.302353](https://doi.org/10.2105/AJPH.2014.302353)
10. Beck AJ, Boulton ML (2016) The Public Health Nurse Workforce in U.S. State and Local Health Departments, 2012. Public Health Rep 131:145–152. doi: [10.1177/003335491613100121](https://doi.org/10.1177/003335491613100121)
11. Beck AJ, Boulton ML, Coronado F (2014) Enumeration of the Governmental Public Health Workforce, 2014. American Journal of Preventive Medicine 47:S306–S313. doi: [10.1016/j.amepre.2014.07.018](https://doi.org/10.1016/j.amepre.2014.07.018)
12. Bjegovic-Mikanovic V, Czabanowska K, Flahault A, et al (2014) Adressing needs in the public health workforce in Europe. European Observatory on Health Systems and Policies, WHO-EURO: Copenhagen, Denmark
13. Blakely T, Pega F, Nakamura Y, et al (2011) Health status and epidemiological capacity and prospects: WHO Western Pacific Region. Int J Epidemiol 40:1109–21
14. Boulton ML, Lemmings J, Beck AJ (2009) Assessment of Epidemiology Capacity in State Health Departments, 2001–2006. Journal of Public Health Management and Practice 15:328. doi: [10.1097/PHH.0b013e3181a01eb3](https://doi.org/10.1097/PHH.0b013e3181a01eb3)
15. Braden KW, Yontz V, Withy K (2017) Preliminary Hawai‘i Public Health Workforce Supply and Demand Assessment. Hawaii J Med Public Health 76:10–14
16. Centers for Disease Control and Prevention (2004) Assessment of Epidemiologic Capacity in State and Territorial Health Departments. <https://www.cdc.gov/mmwr/preview/mmwrhtml/mm5418a2.htm>. Accessed 13 Jun 2018
17. Centers for Disease Control and Prevention (2013) Assessment of Epidemiology Capacity in State Health Departments. <https://www.cdc.gov/mmwr/preview/mmwrhtml/mm6414a6.htm>. Accessed 13 Jun 2018
18. Centers for Disease Control and Prevention (CDC) (2009) Assessment of epidemiology capacity in State Health Departments - United States, 2009. MMWR Morb Mortal Wkly Rep 58:1373–1377
19. Centers for Disease Control and Prevention (CDC) (2011) Food safety epidemiology capacity in state health departments--United States, 2010. MMWR Morb Mortal Wkly Rep 60:1701–4
20. Centers for Disease Control and Prevention (CDC) (2012) The epidemiology workforce in state and local health departments - United States, 2010. MMWR Morb Mortal Wkly Rep 61:205–8
21. Centre for Workforce Intelligence, Royal Society for Public Health (2015) Understanding the wider public health workforce
22. CFWI (2017) Mapping the core public health workforce - final report. United Kingdom
23. Chapman J, Congdon P, Shaw S, Carter YH (2005) The geographical distribution of specialists in public health in the United Kingdom: is capacity related to need? Public Health 119:639–646. doi: [10.1016/j.puhe.2004.10.020](https://doi.org/10.1016/j.puhe.2004.10.020)
24. Chen L-W, Nguyen AT, Jacobson J, Palm D (2012) Assessment of workforce capacity for Local Health Departments in Nebraska: a perspective from public health programmatic areas. J Public Health Manag Pract 18:595–601. doi: [10.1097/PHH.0b013e3182602f1d](https://doi.org/10.1097/PHH.0b013e3182602f1d)
25. Cianciara D, Lewtak K, Piotrowicz M, et al (2016) Public health physicians and dentists in Poland: results from public health workforce pilot study. Rocz Panstw Zakl Hig 67:435–443
26. Coronado F, Polite M, Glynn MK, et al (2014) Characterization of the federal workforce at the Centers for Disease Control and Prevention. J Public Health Manag Pract 20:432–41
27. Council on linkages between academia and public health practice Overall Public Health Workforce. 7
28. Cowles C (2017) Planned and unplanned futures for the Public Health Physician Workforce in Australia. 127
29. Djibuti M, Gotsadze G, Mataradze G, Menabde G (2008) Human resources for health challenges of public health system reform in Georgia. Human Resources for Health 6:8. doi: [10.1186/1478-4491-6-8](https://doi.org/10.1186/1478-4491-6-8)
30. Frank MW, Weihofen A, Schmucki MD, et al (2013) Public Health Workforce in Switzerland: A National Census. Foundation Swiss School of Public Healthplus
31. Frogner BK (2018) Update on the Stock and Supply of Health Services Researchers in the United States. Health Serv Res 53:3945–3966. doi: [10.1111/1475-6773.12988](https://doi.org/10.1111/1475-6773.12988)
32. Gadiel D, Ridoutt L, Lin V, et al (2011) Audit of the Preventive Health Workforce in Australia. Human Capital Alliance
33. Gebbie K, Merrill J, Hwang I, et al (2003) The public health workforce in the year 2000. J Public Health Manag Pract 9:79–86
34. Gray S, Perlman F, Griffiths S (2005) A survey of the specialist public health workforce in the UK in 2003. PUBLIC HEALTH 119:900–906. doi: [10.1016/j.puhe.2005.03.010](https://doi.org/10.1016/j.puhe.2005.03.010)
35. Hassan MA, Akhter S, Shahjahan M (2010) Current population-based public health workforces in Bangladesh. Bangladesh Medical Research Council Bulletin 35:112–113. doi: [10.3329/bmrcb.v35i3.4082](https://doi.org/10.3329/bmrcb.v35i3.4082)
36. Haughton B, George A (2008) The Public Health Nutrition workforce and its future challenges: the US experience. Public Health Nutrition 11:782–791. doi: [10.1017/S1368980008001821](https://doi.org/10.1017/S1368980008001821)
37. Hughes R (2004) Enumerating and profiling the designated public health nutrition workforce in Australia. Nutr Diet 61:162–71
38. Jabbour S, Yamout R (eds) (2012) Public health in the Arab world. Cambridge University Press, Cambridge
39. Jambroes M, Essink-Bot M-L, Plochg T, Stronks K (2012) [Public healthcare occupations--insight into size and composition is limited]. Ned Tijdschr Geneeskd 156:A4529
40. Jambroes M, Lamkaddem M, Stronks K, Essink-Bot ML (2016) [Enumerating the preventive youth health care workforce: size, composition and regional variation in the Netherlands]. Ned Tijdschr Geneeskd 160:D779
41. Jambroes M, van Honschooten R, Doosje J, et al (2015) How to characterize the public health workforce based on essential public health operations? environmental public health workers in the Netherlands as an example. BMC Public Health 15:750. doi: [10.1186/s12889-015-2095-5](https://doi.org/10.1186/s12889-015-2095-5)
42. Jones JA, Banks L, Plotkin I, et al (2015) Profile of the Public Health Workforce: Registered TRAIN Learners in the United States. Am J Public Health 105:e30–e36. doi: [10.2105/AJPH.2014.302513](https://doi.org/10.2105/AJPH.2014.302513)
43. Kennedy VC (2009) Public health workforce employment in US public and private sectors. J Public Health Manag Pract 15:E1-8. doi: [10.1097/01.PHH.0000349744.11738.25](https://doi.org/10.1097/01.PHH.0000349744.11738.25)
44. Leider JP, Coronado F, Beck AJ, Harper E (2018) Reconciling Supply and Demand for State and Local Public Health Staff in an Era of Retiring Baby Boomers. Am J Prev Med 54:334–340. doi: [10.1016/j.amepre.2017.10.026](https://doi.org/10.1016/j.amepre.2017.10.026)
45. Leider JP, Shah GH, Castrucci BC, et al (2014) Changes in public health workforce composition: proportion of part-time workforce and its correlates, 2008-2013. Am J Prev Med 47:S331-336. doi: [10.1016/j.amepre.2014.07.017](https://doi.org/10.1016/j.amepre.2014.07.017)
46. Lenthall S, Wakerman J, Opie T, et al (2011) Nursing workforce in very remote Australia, characteristics and key issues. Aust J Rural Health 19:32–7
47. Li X, Cochran C, Lu J, et al (2015) Understanding the shortage of village doctors in China and solutions under the policy of basic public health service equalization: evidence from Changzhou. Int J Health Plann Manage 30:E42-55
48. Lomazzi M, Wordley V, Bedi R (2016) Dental public health capacity worldwide: Results of a global survey. J Public Health Policy 37:528–542. doi: [10.1057/s41271-016-0029-9](https://doi.org/10.1057/s41271-016-0029-9)
49. Lovell SA, Egan R, Robertson L, Hicks K (2015) Health promotion funding, workforce recruitment and turnover in New Zealand. J Prim Health Care 7:153–157
50. Magaña-Valladares L, Nigenda-López G, Sosa-Delgado N, Ruiz-Larios JA (2009) Public Health Workforce in Latin America and the Caribbean: assessment of education and labor in 17 countries. Salud Pública de México 51:62–75
51. Matrix Insight, Centre for Workforce Intelligence (2012) EU level Collaboration on Forecasting Health Workforce Needs, Workforce Planning and Health Workforce Trends - A Feasibility Study. European Commission
52. McGinnis S, Moore J (2009) The health services research workforce: current stock. Health Serv Res 44:2214–26
53. Medical Council of New Zealand (2016) The New Zealand Medical Workforce in 2016
54. Ministry of Health, Manatu Hauora (2007) Te Uru Kahikatea: The Public Health Workforce Development Plan, 2007 - 2016. Wellington: Ministry of Health
55. Ministry of Helath and Le Va (2012) Taeao o Tautai: Pacific Public Health Workforce Development Implementation Plan. Wellington: Ministry of Health
56. National Association of County and City Health Officials (2005) National Profile of Local Health Departments 2005
57. National Association of County and City Health Officials (2008) National Profile of Local Health Departments 2008
58. National Association of County and City Health Officials (2010) National Profile of Local Health Departments 2010
59. National Association of County and City Health Officials (2013) National Profile of Local Health Departments 2013
60. National Association of County and City Health Officials (2016) National Profile of Local Health Departments 2016
61. Nursing Council of New Zealand (2018) The New Zealand Nursing Workforce: A profile of Nurse Practitioners, Registered Nurses and Enrolled Nurses 2016 - 2017
62. O’Keefe KA, Shafir SC, Shoaf KI (2013) Local health department epidemiologic capacity: a stratified cross-sectional assessment describing the quantity, education, training, and perceived competencies of epidemiologic staff. Front Public Health 1:64. doi: [10.3389/fpubh.2013.00064](https://doi.org/10.3389/fpubh.2013.00064)
63. Qi X, Wang Y, Xia L, et al (2015) Cross-sectional survey on public health informatics workforce in China: issues, developments and the future. Public Health 129:1459–1464. doi: [10.1016/j.puhe.2015.03.002](https://doi.org/10.1016/j.puhe.2015.03.002)
64. Ridoutt L, Gadiel D, Cook K, Wise M (2004) Calculating demand for an effective public health workforce Final Report for the National Public Health Partnership. Human Capital Alliance
65. Rosenblatt RA, Casey S, Richardson M (2002) Rural–Urban Differences in the Public Health Workforce: Local Health Departments in 3 Rural Western States. Am J Public Health 92:1102–1105
66. Rosenstock L, Silver GB, Helsing K, et al (2008) On Linkages: Confronting the Public Health Workforce Crisis: Asph Statement on the Public Health Workforce. Public Health Rep 123:395–398
67. Russell ML, McIntyre L (2009) An Estimation of Canada’s Public Health Physician Workforce. Canadian Journal of Public Health; Ottawa 100:199–203
68. Santric Milicevic M, Vasic M, Edwards M, et al (2018) Strengthening the public health workforce: An estimation of the long-term requirements for public health specialists in Serbia. Health Policy. doi: [10.1016/j.healthpol.2018.03.012](https://doi.org/10.1016/j.healthpol.2018.03.012)
69. Sommanustweechai A, Putthasri W, Nwe ML, et al (2016) Community health worker in hard-to-reach rural areas of Myanmar: filling primary health care service gaps. Hum Resour Health 14:64
70. Tiwari R, Negandhi H, Zodpey SP (2018) Health Management Workforce for India in 2030. Front Public Health 6:227
71. Tomar SL (2006) An assessment of the dental public health infrastructure in the United States. J Public Health Dent 66:5–16
72. Turcanu G, Domente S, Buga M, Richardson E (2012) Republic of Moldova health system review. Health Syst Transit 14:1–151
73. Underwood JM, Mowat DL, Meagher-Stewart DM, et al (2009) Building community and public health nursing capacity: a synthesis report of the National Community Health Nursing Study. Can J Public Health 100:I1-11
74. United States Department of Agriculture - Food and Nutrition Service (2003) Survey of the Public Health Nutrition Workforce: 1999-2000
75. University of Michigan Center of Excellence in Public Health Workforce Studies (2013) Public Health Workforce Enumeration, 2012. Ann Arbor, MI: University of Michigan
76. University of Michigan Center of Excellence in Public Health Workforce Studies, Association of Public Health Laboratories (2012) National Laboratory Capacity Assessment, 2011
77. US Department of Health and Human Services - Health Resources and Services Administration (2005) Public Health Workforce
78. Wong ST, Watson DE, Young E, Mooney D (2009) Supply and distribution of primary healthcare registered nurses in british columbia. Healthc Policy 5 Spec no:91–104
79. World Health Organization (2016) Joint External Evaluation of IHR Core Capacities - State of Eritrea. Geneva
80. World Health Organization, Regional Office for Europe, European Observatory on Health Systems and Policies (2018) Organization and financing of public health services in Europe: Country Reports. Brussels
81. Yin D, Yin T, Yang H, et al (2016) An economic-research-based approach to calculate community health-staffing requirements in Xicheng District, Beijing. Hum Resour Health 14:. doi: [10.1186/s12960-016-0152-5](https://doi.org/10.1186/s12960-016-0152-5)
82. Unknown, Number of physicians and physicians/100,000 population in Public Health and Preventive Medicine in Canada, 2015: https://legacy.cma.ca//Assets/assets-library/document/en/advocacy/Public-Health-e.pdf
