# Supplementary material for: No Two Workforces Are the Same: A Systematic Review of Enumerations and Definitions of Public Health Workforces
Source: Front Public Health. 2020 Nov 19;8:588092. doi: 10.3389/fpubh.2020.588092 (PMC7711128; doi:10.3389/fpubh.2020.588092)
Supplement: Supplementary file 2 [file Data_Sheet_1.docx]

Supplement - Search Strategy and Yield for databases

|  | **Web of Science** | **PubMed** | **ProQuest** | **Google Scholar*** | **ScienceDirect*** |
| --- | --- | --- | --- | --- | --- |
| **#1** | 2,199 | 4,156 | 3,418 | 200 | 200 |
| **#2** | 284 | 2,521 | 2,291 |  |  |
| **#3** | 27 |  |  |  |  |
| **#4** | 2,494 |  |  |  |  |
| **Total** |  |  |  |  | *17,790* |

Table 1 - Search yield by strategy by database. Total does not exclude duplicates. * Limited to 200 results

| **Strategy** | **String** | **Combine above with** |
| --- | --- | --- |
| #1 | ((population health[Title/Abstract])OR(public health[Title/Abstract]))AND( (enumer*[Title/Abstract])OR (workforce[Title/Abstract])OR (demand[Title/Abstract])OR (supply[Title/Abstract])) |  |
| #1 | (("Dentists"[Mesh] AND "public health") OR("Environmental Health"[Mesh]) OR("Community Medicine"[Mesh]) OR("Public Health Nursing"[Mesh]) OR("Epidemiologists"[Mesh]) OR("Physicians"[Mesh] AND "public health") OR(Epidemiology[Mesh]) OR(Preventive Medicine[Mesh]) OR("Public Health"[Mesh]) OR("Global Health"[Mesh]) OR("Public Health Dentistry"[Mesh]) OR("biostatistician") OR("Health promotion") OR)AND((enumerat*) OR(demand) OR(supply) OR(Forecasting[Mesh]) OR("Health Services Needs and Demand"[Mesh]) OR("Forecasting") OR("numbers") OR)AND(("Workforce"[Title/Abstract]) OR("Health Workforce"[Mesh]) OR("Workforce"[Mesh]) OR)) | OR |
| #1 | ("public health" AND "workforce") | OR |
| #1 | ( "Health Workforce/economics"[Mesh] OR "Health Workforce/statistics and numerical data"[Mesh] OR "Health Workforce/supply and distribution"[Mesh] OR "Health Workforce/trends"[Mesh] ) | OR |
| #1 | (("address"[Publication Type] OR "autobiography"[Publication Type] OR "bibliography"[Publication Type] OR "biography"[Publication Type] OR "book illustrations"[Publication Type] OR "comment"[Publication Type] OR "dataset"[Publication Type] OR "dictionary"[Publication Type] OR "directory"[Publication Type] OR "editorial"[Publication Type] OR "expression of concern"[Publication Type] OR "interview"[Publication Type] OR "legal case"[Publication Type] OR "legislation"[Publication Type] OR "letter"[Publication Type] OR "news"[Publication Type] OR "newspaper article"[Publication Type] OR "personal narrative"[Publication Type] OR "pictorial work"[Publication Type] OR "popular work"[Publication Type] OR "portrait"[Publication Type] OR "research support, american recovery and reinvestment act"[Publication Type] OR "research support, n i h, extramural"[Publication Type] OR "research support, n i h, intramural"[Publication Type] OR "research support, non u s gov't"[Publication Type] OR "research support, u s gov't, non p h s"[Publication Type] OR "research support, u s gov't, p h s"[Publication Type] OR "research support, u s government"[Publication Type] OR "support of research"[Publication Type])) | AND |
| #2 | ((population health[Title])OR(public health[Title])OR(epidemiol*[Title])OR(health science[Title])OR(biostat*[Title])OR(health promotion[Title]))AND((capacity[Title])OR (job*[Title])OR (skill*[Title])OR (train*[Title])OR (enumer*[Title])OR (educat*[Title])OR (workforce[Title])OR (manpower[Title])OR (human resource*[Title])OR (demand[Title])OR (worker*[Title])OR (occupation*[Title])OR (tasks[Title])OR (duties[Title])OR (labour[Title])OR (labor[Title])OR (gradu*[Title])OR (supply[Title])) |  |

Table 2: PubMed search strategies

| **Strategy** | **String** |
| --- | --- |
| #1 | (All=((“public health”) OR (“population health”)) AND All=(“workforce”)) |
| #2 | (TS=(“PUBLIC HEALTH WORKFORCE’)) |
| #3 | ((TS=(“ENUMERATION”) AND TS=(“WORKFORCE”))) |
| #4 | ((TI= ((capacity) OR (job*) OR (skill*) OR (train*) OR (enumer*) OR (workforce) OR (labor) OR (labour) OR (occupation) OR (tasks) OR (duties) OR (worker*) OR (manpower) OR (human resource*) OR (demand) OR (supply)))) AND ((TI=((population health) OR (public health) OR (epidemiol*) OR (biostat*) OR (health promotion) OR (health science) OR (gradu*)))) |

Table 3: Web of Science search strategies

| #1 | (ti(population health) OR ti(public health)OR ti(epidemiol*)OR ti(biostat*)OR ti(health promotion)OR ti(health science))AND( ti(capacity)OR ti(job*)OR ti(skill*)OR ti(train*)OR ti(enumer*)OR ti(educat*)OR ti(workforce)OR ti(manpower)OR ti(human resource*)OR ti(worker*)OR ti(occupation*)OR ti(tasks)OR ti(duties)OR ti(labour)OR ti(labor)OR ti(demand)OR ti(supply)OR ti(gradu*)) |
| --- | --- |
| #2 | (ti("public health") OR ab("public health")) AND (ti("workforce") OR ab("workforce")) |

Table 4: ProQuest search strategies
