# Supplementary material for: No Two Workforces Are the Same: A Systematic Review of Enumerations and Definitions of Public Health Workforces
Source: Front Public Health. 2020 Nov 19;8:588092. doi: 10.3389/fpubh.2020.588092 (PMC7711128; doi:10.3389/fpubh.2020.588092)
Supplement: Supplementary file 3 [file Data_Sheet_2.docx]

ISCO-08 Mapping Procedure

We mapped occupations from publications to the ISCO-08 list of occupations. We did this in a two-step process.

1. Collected all occupations which were mentioned
2. Used our judgement to map these to the ISCO-08 occupational categories

# Collected all occupations which were mentioned

"academic public health specialists", "administrators", "agency leadership", "allied health professional", "animal control", "assistant health inspector", "behavioral health professional", "behavioral health staff", "biologists","biostatisticians", "business operations", "chemists", "community health nurses", "community health workers", "community health workers", "coordinators", "dental therapists", "dentist", "dentists", "dieticians", "directors of public health", "disinfectors", "doctor", "economists", "emergency preparedness staff", "employees at local health agencies", "engineers", "environmental health experts", "environmental public health workers", "epidemiologists", "family doctors", "financial operations", "fitness instructors", "food inspectors", "food safety epidemiologists", "health assistant", "health care administrators", "health care educators", "health economist", "health engineers", "health inspector", "health inspectors", "health management", "health planners", "health policy makers", "health prevention workers", "health promoters", "health promotion managers", "health promotion specialists", "health promotion", "health protection officer", "health services researchers", "health technicians", "health visitors", "hygienists", "IT workers", "lab technicians", "laboratory assistant", "laboratory worker", "licensed practical or vocational nurse", "managers", "microbiology", "midwives", "midwives", "nurses", "nurses", "nursing technicians", "nursing home health aide", "nutritionists", "nutritionists", "nutritionists", "occupational and environmental health specialists", "office support staff", "oral health care", "parasitology", "pharmacists", "physicians", "physician assistants", "policy analysts", "preventive youth healthcare", "primary healthcare registered nurses", "psychologists", "psychologists", "public health consultants", "public health dental worker", "public health dieticians", "public health informatics", "public health information specialist", "public health manager", "public health nurse", "public health nutritionists", "public health physicians", "public health practitioners", "public health scientists", "public health specialists", "public health workers", "quality improvement specialist", "registered nurses", "researchers", "sanitary engineers", "school nurses", "scientific laboratory staff", "social medicine", "social nursing", "social scientist", "social workers", "sociologists", "sociomedical assistants", "specialist doctors", "statisticians", "typists", "village doctors"

# Mapped these to ISCO-08 occupational categories

Below we list the contents of a dictionary used to map the occupations in Section 1 with their corresponding ISCO-08 occupation. The dictionary takes the form of

KEY : {‘isco-8 mapping’: VALUE}

Where the KEY is the original occupation, and VALUE is the ISCO-08 mapping. Some elements contain a #Comment, which we used to document any uncertainty around the mapping. We used the Group Definitions PDF and Correspondence Tables at this link: <https://www.ilo.org/public/english/bureau/stat/isco/isco08/>

'IT workers': {'isco-8 mapping': 2511},

'academic public health specialists': {'isco-8 mapping': 2310},

'administrators': {'isco-8 mapping': 3343},

'agency leadership': {'isco-8 mapping': 1342},

'allied health professional': {'isco-8 mapping': 2269},

'animal control': {'isco-8 mapping': 2250},

'assistant health inspector': {'isco-8 mapping': 3257},

'behavioral health professional': {'isco-8 mapping': 2635},

'behavioral health staff': {'isco-8 mapping': 2635},

'biologists': {'isco-8 mapping': 2131},

'biostatisticians': {'isco-8 mapping': 2120},

'business operations': {'isco-8 mapping': 1219},

'chemists': {'isco-8 mapping': 2113},

'community health nurses': {'isco-8 mapping': 2221},

'community health workers': {'isco-8 mapping': 3253},

'coordinators': {'isco-8 mapping': 1342},

'dental therapists': {'isco-8 mapping': 2261},

'dentist': {'isco-8 mapping': 2261},

'dieticians': {'isco-8 mapping': 2265},

'directors of public health': {'isco-8 mapping': 1342},

'disinfectors': {'isco-8 mapping': 9112},

'doctor': {'isco-8 mapping': 2212},

'economists': {'isco-8 mapping': 2631},

'emergency preparedness staff': {'isco-8 mapping': 0}, #Comment: We weren’t confident in any classification of this ‘occupation’

'employees at local health agencies': {'isco-8 mapping': 0}, #Comment: We weren’t confident in any classification of this ‘occupation’

'engineers': {'isco-8 mapping': 2143}, #Comment: Have assumed they are referring to environmental / health engineers

'environmental health experts': {'isco-8 mapping': 2263},

'environmental public health workers': {'isco-8 mapping': 2263},

'epidemiologists': {'isco-8 mapping': 2131}, #Comment: index08-draft.xlsx labels epidemiologist in this category, despite not appearing in the documentation specifically

'family doctors': {'isco-8 mapping': 2211},

'financial operations': {'isco-8 mapping': 1211},

'fitness instructors': {'isco-8 mapping': 3423},

'food inspectors': {'isco-8 mapping': 3257},

'food safety epidemiologists': {'isco-8 mapping': 2131}, #Comment: opted for labelling as epidemiologist

'health assistant': {'isco-8 mapping': 0},

'health care administrators': {'isco-8 mapping': 1342},

'health care educators': {'isco-8 mapping': 3253}, #Comment: no specific mapping, so mapped to Community Health Worker

'health economist': {'isco-8 mapping': 2631},

'health engineers': {'isco-8 mapping': 2143}, #Comment: Mapped to environmental engineer as it seemed the closest fit

'health inspector': {'isco-8 mapping': 3257},

'health management': {'isco-8 mapping': 1342},

'health planners': {'isco-8 mapping': 1342},

'health policy makers': {'isco-8 mapping': 1342},

'health prevention workers': {'isco-8 mapping': 3253}, #Comment: Mapped to CHW

'health promotion': {'isco-8 mapping': 3253}, #Comment: Mapped to CHW because of CH promoter

'health promotion managers': {'isco-8 mapping': 1342},

'health promotion specialists': {'isco-8 mapping': 3253},

'health protection officer': {'isco-8 mapping': 0}, #Comment: Did not map this as it seemed too ambiguous

'health services researchers': {'isco-8 mapping': 2131}, #Comment: Stuck with 2131 which is a biomedical researcher. Also the code for epidemiologist

'health technicians': {'isco-8 mapping': 0}, #Comment: Did not map this, ambiguous

'health visitors': {'isco-8 mapping': 2221},

'hygienists': {'isco-8 mapping': 3257}, #Comment: 'assuming similar to a sanitarian'

'lab technicians': {'isco-8 mapping': 3212},

'laboratory assistant': {'isco-8 mapping': 3212},

'laboratory worker': {'isco-8 mapping': 3212},

'licensed practical or vocational nurse': {'isco-8 mapping': 2221},

'managers': {'isco-8 mapping': 1342},

'microbiology': {'isco-8 mapping': 2131},

'midwives': {'isco-8 mapping': 2222},

'nurses': {'isco-8 mapping': 2221},

'nursing home health aide': {'isco-8 mapping': 2221},

'nursing technicians': {'isco-8 mapping': 2221},

'nutritionists': {'isco-8 mapping': 2265},

'occupational and environmental health specialists': {'isco-8 mapping': 2263},

'office support staff': {'isco-8 mapping': 3343},

'oral health care': {'isco-8 mapping': 2261},

'parasitology': {'isco-8 mapping': 2131},

'pharmacists': {'isco-8 mapping': 2262},

'physician assistants': {'isco-8 mapping': 2211}, #Comment: Have classified as medical doctor due to functions performed

'physicians': {'isco-8 mapping': 2211},

'policy analysts': {'isco-8 mapping': 2422},

'preventive youth healthcare': {'isco-8 mapping': 3412}, #Comment: Classified as social work

'primary healthcare registered nurses': {'isco-8 mapping': 2221},

'psychologists': {'isco-8 mapping': 2634},

'public health consultants': {'isco-8 mapping': 2212},

'public health dental worker': {'isco-8 mapping': 2261},

'public health dieticians': {'isco-8 mapping': 2265},

'public health informatics': {'isco-8 mapping': 3252},

'public health information specialist': {'isco-8 mapping': 3252},

'public health manager': {'isco-8 mapping': 1342},

'public health nurse': {'isco-8 mapping': 2221},

'public health nutritionists': {'isco-8 mapping': 2265},

'public health physicians': {'isco-8 mapping': 2212},

'public health practitioners': {'isco-8 mapping': 2212}, #Comment: Have assumed this is a medical doctor speciality

'public health scientists': {'isco-8 mapping': 2131}, #Comment: No particularly good mapping, stuck with 2131 like epidemiologist

'public health specialists': {'isco-8 mapping': 2212},

'public health workers': {'isco-8 mapping': 3253}, #Comment: Classified as CHW

'quality improvement specialist': {'isco-8 mapping': 0}, #Comment: Did not classify, ambiguous

'registered nurses': {'isco-8 mapping': 2221},

'researchers': {'isco-8 mapping': 2131},

'sanitary engineers': {'isco-8 mapping': 2143},

'school nurses': {'isco-8 mapping': 2221},

'scientific laboratory staff': {'isco-8 mapping': 3212},

'social medicine': {'isco-8 mapping': 2211},

'social nursing': {'isco-8 mapping': 2221},

'social scientist': {'isco-8 mapping': 2632},

'social workers': {'isco-8 mapping': 2635},

'sociologists': {'isco-8 mapping': 2632},

'sociomedical assistants': {'isco-8 mapping': 0}, #Comment: Did not classify, ambiguous

'spcialist doctors': {'isco-8 mapping': 2212},

'statisticians': {'isco-8 mapping': 2120},

'typists': {'isco-8 mapping': 4131},

'village doctors': {'isco-8 mapping': 3253}
