# Supplementary figures and images for: No Two Workforces Are the Same: A Systematic Review of Enumerations and Definitions of Public Health Workforces
Source: Front Public Health. 2020 Nov 19;8:588092. doi: 10.3389/fpubh.2020.588092 (PMC7711128; doi:10.3389/fpubh.2020.588092)

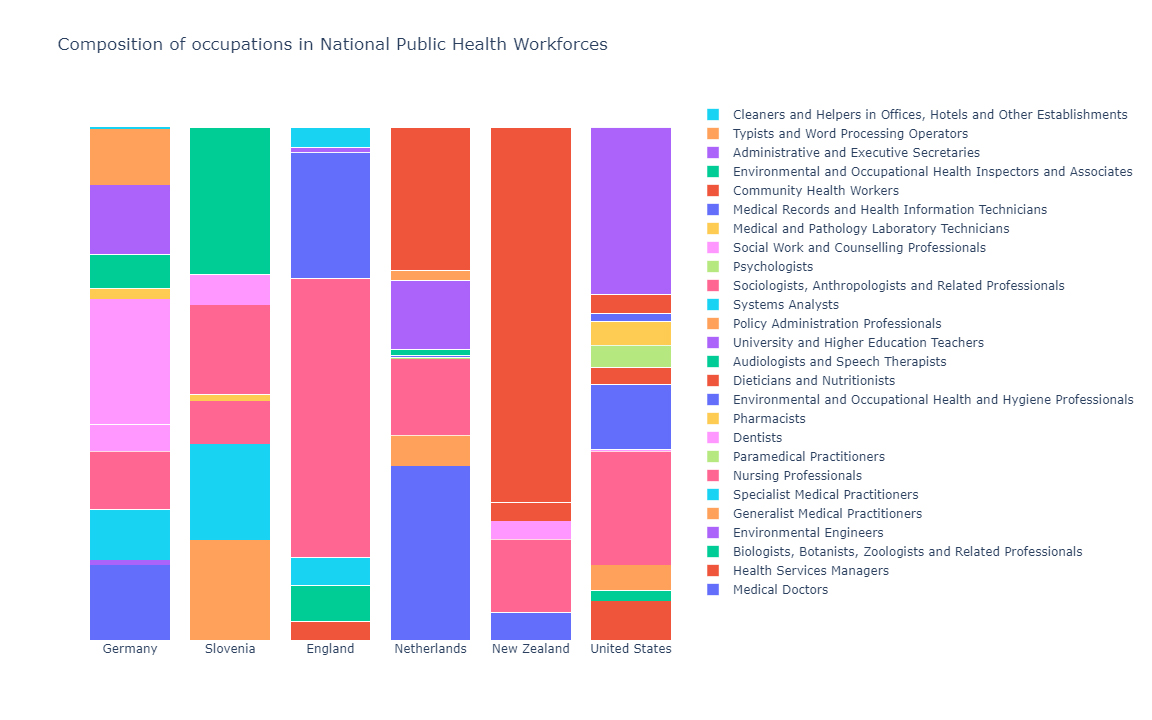

Supplement: Supplementary file 4 [file Image_1.JPEG]
